# *Angelica gigas* ameliorates the destruction of gingival tissues *via* inhibition of MMP-9 activity†

**DOI:** 10.1039/c7ra12531c

**Published:** 2018-04-09

**Authors:** Mi Hye Kim, Haesu Lee, Woong Mo Yang

**Affiliations:** Department of Convergence Korean Medical Science, College of Korean Medicine, Kyung Hee University Seoul 02447 Republic of Korea wmyang@khu.ac.kr +82-2-961-2209 +82-2-961-2209

## Abstract

*Angelica gigas* (AG) has been used for periodontal diseases in traditional Korean medicine. However, the effects of AG on periodontitis have not been clarified yet. In this study, we investigated the ameliorative effects of AG against ligature-induced periodontitis. Sprague-Dawley rats were divided into four groups; non-ligatured (normal), ligatured and treated with vehicle (ligatured), ligatured and treated with 1 mg mL^−1^ AG (AG1), and ligatured and treated with 100 mg mL^−1^ AG (AG100). 70% ethanol extracts of AG were topically applied onto both sides of the first molar daily for 14 days. In addition, human dermal fibroblast cells were treated with 1, 10 and 100 μg mL^−1^ AG to characterize the expression of matrix metallopeptidase 9 (MMP-9). Topical AG treatment reduced alveolar bone resorption, as assessed by methylene blue staining. The structures of soft gingival tissues (periodontal pocket) were recovered in the AG-treated groups. The expression of MMP-9 was decreased, and that of type 1 collagen was significantly increased in AG-treated gingival tissues. In addition, AG treatment inhibited the activity of MMP-9 in LPS-treated human dermal fibroblast cells. This study reveals that topical AG treatment has the potential to ameliorate the destruction of gingival tissues by inhibiting MMP-9 activity. AG may be a candidate for the treatment of periodontitis.

## Abstract 2

*Angelica gigas* (AG) has been used for periodontal diseases in traditional Korean medicine.

## Graphical abstract

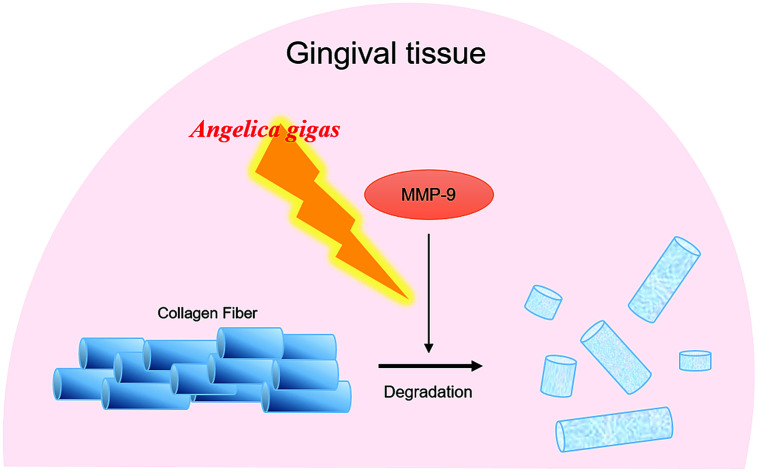

## 1. Introduction

Periodontal disease is caused by specific oral microbes that infect up to 20% of the global adult population.^1^ Typical consequences of periodontitis include alveolar bone resorption, gingival inflammation, periodontal pocket formation and the breakdown of supporting connective tissues, ultimately resulting in tooth loosening from the jaw bone.^2^

To date, several periodontal treatments have focused on modulation of the host immune response. Subantimicrobial dose doxycycline, a host response modifier, is an approved systemic therapy that decreases prostaglandins and inflammatory cytokines. However, accelerated bone loss sometimes occurs after cessation of treatment.^3,4^ Recently, a combination of host modulatory therapy involving implantation, chemical root conditioning and growth factors has been used in clinical trials to reconstruct periodontal tissues.^5^ However, these therapies have limitations related to adverse effects and high costs; therefore, identification of alternative treatments for periodontal diseases is still needed.

Traditionally, the dried roots of *A. gigas* Nakai (Apiaceae) have been used as a functional food and herbal medicine to enrich and tonify the blood for thousands of years.^6^ Several studies have demonstrated its pharmacological activities, which include inhibition of cancer, bacterial and nematode infection, platelet aggregation and oxidation.^7^ Especially, *A. gigas* was reported to be used as either formula or single for dental health based on the classic book.^8^ Previous studies demonstrate that *A. gigas* includes various chemical components such as decursin, decursinol, decursinol angelate, nodakenin, *n*-butylidenephthalide, and umbelliferoneate. Decursin has been used as a standard of identification of *A. gigas*, as a main bioactive compound of AG.^9^ Recently, decursin has the potential to inhibit ultraviolet B-induced matrix metalloproteinase (MMP)-1 and MMP-3 expression in human dermal fibroblast (HDF) cells.^10^ Decursin has also been shown to ameliorate bone loss by inhibiting osteoclastogenesis.^11^ However, there is still a lack of study on the effects and underlying mechanism of *A. gigas* on periodontitis. In this study, we investigated the effects of *A. gigas* on periodontitis and potential mechanism in rats.

## 2. Experimental

### 2.1. Preparation of AG extract

Thirty grams of dried roots of *A. gigas* Nakai (Jung-do Herb; Seoul, Korea) was extracted with 300 mL of 70% ethanol for 24 h at room temperature. The extract was filtered with Whatman filter paper no. 3 (Whatman, Maidstone, Kent, England), concentrated in a rotary vacuum evaporator and freeze-dried. The weight of final dried powder (named AG) was 11.27 g (yield: 37.6%). A voucher specimen (AG070, 70% ethanol extract of *A. gigas*) was deposited at our laboratory.

Decursin, which is main component of *A. gigas*, was used to identify AG by High-Performance Liquid Chromatography-Evaporative Light Scattering Detector (HPLC-ELSD Agilent 1100 series). The extract was dissolved in 70% methanol and sonicated 30 min. After filtering, aliquot mixed with methanol was injected in HPLC analysis. The used column was SHISEIDO CAPCELL PAK C18 (250 × 4.6 mm, 5 μm). The mobile phase consisted of 30 mM ammonium acetate and acetonitrile (20 : 80) with 1.0 mL min^−1^ of flow rate at 30 °C. The peak of decursin in AG was synchronized with standard decursin.

### 2.2. Experimental design

Twenty-eight male Sprague Dawley rat aged 7 weeks (RaonBio Inc., Yongin, Korea) were adapted for 1 weeks in an air-conditioned room under a 12 h light/dark cycle with food and water freely available. Appropriate temperature and humidity were maintained for animal's convenience. All experiments were approved by Committee on Care and Use of Laboratory Animals of the Kyung Hee University (KHUASP(SE)-14-029).

Twenty one rats were ligatured with sterilized 3–0 nylon into the subgingival sulcus around the both sides of first mandibular molar of rats while the rest of rats were non-ligatured, and randomly divided into 4 groups (*n* = 7, respectively); (normal) no ligature placement and non-treatment, (ligatured) ligature placement and administration of vehicle, (AG1) ligature placement and administration of AG 1 mg mL^−1^ and (AG100) ligature placement and administration of AG 100 mg mL^−1^. For reliability of topical application, 1% carboxymethylcellulose was added to AG solution in distilled water based on previous report.^12^ AG was administered once daily in 100 μL of volume at 1 and 100 mg mL^−1^ for consecutive 14 days. Then, rats were sacrificed.

### 2.3. Measurement of mandible bone loss

The right side of the mandibles were collected and immersed in 1% methylene blue aqueous solution (Sigma, MO, USA) for 1 min at room temperature and 20 ± 5% humidity. After drying with compressed air, the specimens were photographed with 100 mm macrolens Canon digital camera (Canon, Tokyo, Japan). The length of three root surfaces from first mandibular molar were measured by a computerized densitometry system Image J (NIH, Bethesda, MD, USA). The score of alveolar bone loss was determined by sum of three values of the length of three root surfaces from first mandibular molar.

### 2.4. Evaluation of histological changes

The left side of the mandibles were fixed in 10% neutral buffered formalin for 18 h and demineralized in a solution of 0.1 M ethylene diamine tetraacetic acid for 2 months. After dehydration with ethanol and xylene, the specimens were embedded in paraffin. Serial sections of 7 μm thickness were obtained and stained with hematoxylin and eosin (H&E). The digital images were obtained from Leica Application Suite (LAS) microscope software (Leica Microsystems, Buffalo Grove, IL, USA) with the ×40 magnification.

### 2.5. Measurement of pro-collagen type 1 and MMP-9

The gingival tissues around the ligature placement were excised. Total RNA from gingival tissues was extracted by Trizol methods. Complementary DNA (cDNA) was synthesized by commercially available cDNA synthesis kits (Invitrogen, Carlsbad, CA, USA) at 45 °C for 60 min and then at 95 °C for 5 min. Reverse transcription polymerase chain reaction (RT-PCR) was performed using 10 μg of cDNA with pre-mixed PCR-kit (Invitrogen). The following primers were used: type 1 collagen, 5′-TCT ACT GGC GAA ACC TGT ATC CG-3′ (forward) and 5′-CAA GGA AGG GCA GGC GTG AT-3′ (reverse). MMP-9, 5′-GGG ACG CAG ACA TCG TCA TC-3′ (forward) and 5′-TCG TCA TCG TCG AAA TGG GC' (reverse). GAPDH, 5′-CCA TCA CCA TCT TCC AGG AG -3′ (forward) and 5′-CCT GCT TCA CCA CCT TCT TG-3′ (reverse). The amplification program was comprised of the initial denaturation step 35 cycles. Relative quantification on gene expression was performed in relation to GAPDH mRNA expression by a computerized densitometry system Image J.

### 2.6. Determination of MMP-9 release level

HDF cells were grown in Dulbecco's modified Eagle's medium supplemented with 10% fetal bovine serum and 1% antibiotics (Gibco-BRL, Grand Island, NY, USA) at 5% CO_2_ and 95% humidity condition. Seeded cells in 6 well plates were infected with 15 μg mL^−1^ of lipopolysaccharide (LPS) and co-treated with 1, 10 and 100 μg mL^−1^ of AG for 24 h. The supernatants were collected and centrifuged. Commercial Human MMP-9 Quantikine enzyme-linked immunosorbent assay kit (R&D systems, USA) was used to analyze the MMP-9 concentration.

### 2.7. Statistical analysis

Significance was determined by one-way analysis of variance (ANOVA) and Dunnett's multiple comparison tests using a GRAPHPAD PRISM 5 software (Ver. 5, GraphPad Software, Inc., CA, USA). In all analyses, *p* < 0.05 was taken to indicate statistical significance.

## 3. Results and discussion

**Fig. 1:**
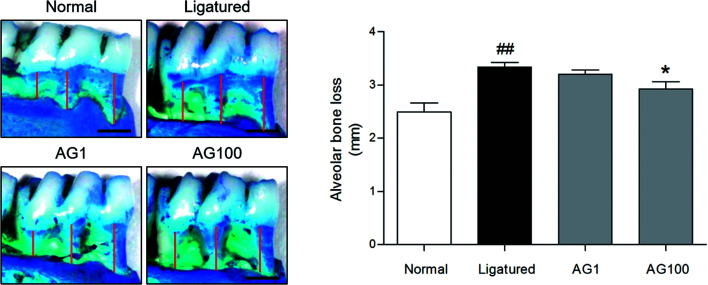
Representative images and quantified values of alveolar bone loss. The scale bar is 1000 μm. The red lines represent the distance from the cementoenamel junction to the alveolar bone crest.

**Fig. 2:**
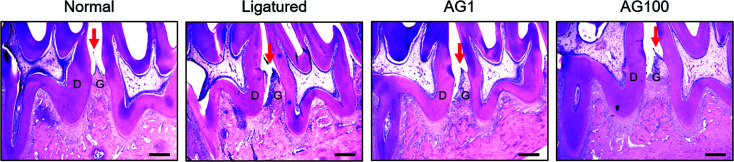
Histological changes of periodontium. The scale bar is 400 μm. Mean values were significantly different for the following comparisons. ^##^*p* < 0.01 compared to normal group; * < 0.05 compared to ligatured group. Red arrows indicate to gingiva. D, dentin; G, gingiva.

Periodontal disease can lead to periodontitis and gingivitis depending on the disease stage.^13^ Gum tissue and connective tissue becomes inflamed (gingivitis) at early stage, and further advances to periodontitis such as alveolar bone loss.^1^ Accordingly, recent therapies for periodontal disease are required to target not only alveolar bone resorption, but also soft tissue degradation.^14^ In the present study, a significant increase of alveolar bone loss was appeared (normal = 2.495 ± 0.34 mm; ligatured = 3.338 ± 0.172 mm) in ligature-induced periodontitis group. Compared to ligatured group, AG 100 mg mL^−1^ treated group showed a significant decrease in alveolar bone loss (AG1 = 3.203 ± 0.167 mm; AG100 = 2.925 ± 0.275 mm; *p* < 0.05, Fig. 1). In addition, severe destruction of gingival tissues in space between first and second mandibles regarded as periodontal pocket was observed in ligatured group in comparison with normal group. Treatment of 1 and 100 mg mL^−1^ of AG recovered the collapsed gingival tissues compared with ligatured group (Fig. 2). Especially, 100 mg mL^−1^ of AG-treated group showed an almost complete recovery nearby normal group. Taken together, AG treatment ameliorated the formation of periodontal pockets resulting from soft tissue destruction, as well as alveolar bone loss, in a ligature-induced periodontitis rat model.

**Fig. 3:**
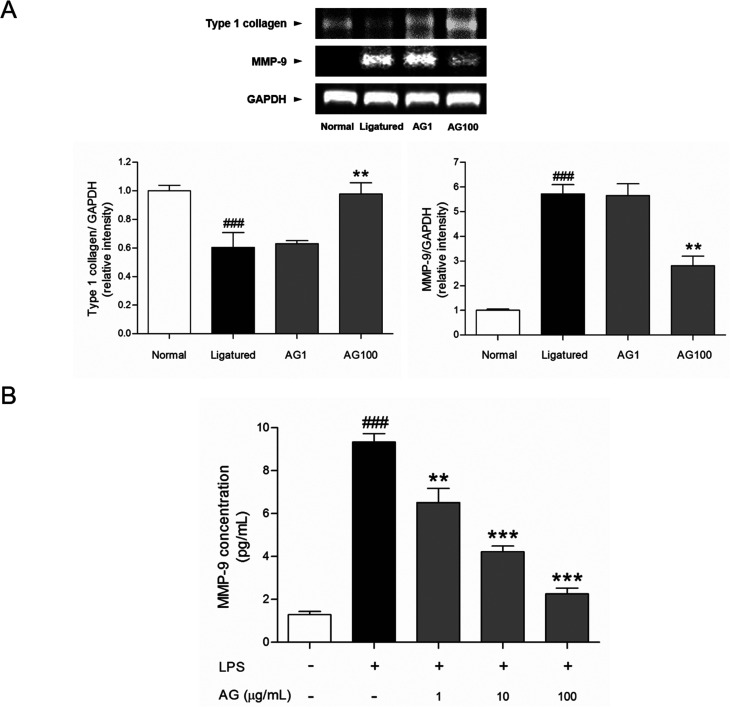
Expressions of type 1 collagen and MMP-9 mRNA in gingival tissues (A). Mean values were significantly different for the following comparisons. ^###^*p* < 0.001 compared to normal group; ** < 0.01 and *** < 0.001 compared to ligatured group. MMP-9 concentration (pg mL^−1^) in LPS-sensitized HDF cells (B). ^###^*p* < 0.001 compared to non-treated group; ** < 0.01 and *** < 0.001 compared to LPS-treated group.

Fibroblasts, which produce a collagen-rich extracellular matrix, are the predominant components of gingival tissues.^15^ Well-organized type 1 collagen is responsible for the adherence of the gingiva to the teeth.^16^ It is well established that gingival inflammation (*i.e.*, gingivitis) accompanies the development of periodontitis. In periodontitis, the destruction of gingival tissues results in the degradation of collagen, including type 1 collagen.^17^ AG treatment recovered the destruction of gingival tissue, which is affected by periodontitis. These improvement of soft tissue inflammation was accompanied with an increase of type 1 collagen mRNA expression by AG treatment. Gingival tissue from ligatured group exhibited a significant lower mRNA expression of type 1 collagen (*p* < 0.05) compared with normal group. The mRNA expressions of type 1 collagen were increased dose-dependently by AG treatment (Fig. 3A).

To clarify the protective effects of AG on collagen degradation, collagenase activity was investigated in gingival tissue. MMPs are responsible for the degradation of denatured interstitial collagens in damaged tissue.^18–20^ As described in previous reports, MMP-9 distributed in fibroblasts, keratinocytes, endothelial cells and osteoblasts could be a key enzyme to disassemble the type 1 collagen. Therefore, inhibition of MMP-9 activity might be involved in ability to attenuate the degradation of gingival tissues and the expression of MMP-9 is seriously increased in impaired gingiva by periodontal inflammation.^21^ The present study shows that the activity of MMP-9 in gingival tissue was significantly inhibited by AG treatment. The mRNA expression of MMP-9 was clearly increased in ligatured group. Treatment of AG 100 mg mL^−1^ reduced the mRNA expression of MMP-9 (Fig. 3A). To confirm the effect on MMP-9 expression by AG treatment, collagenase activity was analyzed *in vitro*. Approximately 7.25 times of increase of MMP-9 level was found in LPS-induced HDF cells. Following treatment with 1, 10 and 100 μg mL^−1^ of AG, elevated MMP-9 concentration were suppressed in dose-dependent manner (30.22, 54.84 and 75.83%, respectively, Fig. 3B). These results suggest that the restoration of gingival tissue by AG treatment is positively correlated with the inhibition of MMP-9 activity.

## 4. Conclusion

**Fig. 4:**
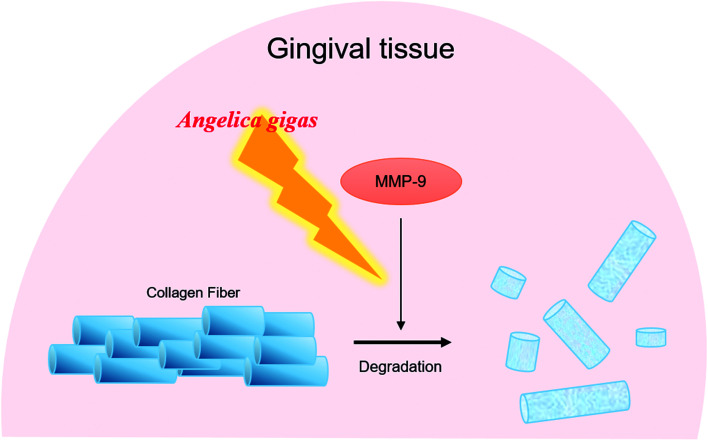
Diagram illustrates the potential actions of AG on destruction of gingival tissues in periodontitis.

In conclusion, topical AG treatment inhibited the alveolar bone loss in ligature-induced periodontitis rat. AG improved the periodontal pocket formation by recovery of the destruction of gingival tissues. The expressions of type 1 collagen and MMP-9 in gingival tissue were significantly regulated by AG administration. The present study revealed that topical AG treatment ameliorates alveolar bone loss and gingiva tissue degradation by inhibiting MMP-9 activity (Fig. 4). These results suggest that AG may help regenerate impaired gingiva in periodontitis.

## Conflicts of interest

The authors declare no conflict of interest.

## Supplementary Material

RA-008-C7RA12531C-s001

## References

[cit1] Offenbacher S. (1996). Annals of periodontology.

[cit2] Williams R. C., Barnett A. H., Claffey N., Davis M., Gadsby R., Kellett M., Lip G. Y., Thackray S. (2008). Curr. Med. Res. Opin..

[cit3] Howell T. H., Williams R. C. (1993). Crit. Rev. Oral Biol. Med..

[cit4] Preshaw P. M., Hefti A. F., Jepsen S., Etienne D., Walker C., Bradshaw M. H. (2004). J. Clin. Periodontol..

[cit5] Yang H., Wen Q., Xue J., Ding Y. (2014). J. Periodontal Res..

[cit6] Cho J. H., Kwon J. E., Cho Y., Kim I., Kang S. C. (2015). Nutrients.

[cit7] Lee B., Sur B., Shim I., Lee H., Hahm D. H. (2015). BMC Complementary Altern. Med..

[cit8] Song B. K., Won J. H., Kim S. (2016). J. Pharmacopuncture.

[cit9] Jeong S. Y., Kim H. M., Lee K. H., Kim K. Y., Huang D. S., Kim J. H., Seong R. S. (2015). Chem. Pharm. Bull..

[cit10] Hwang B. M., Noh E. M., Kim J. S., Kim J. M., Hwang J. K., Kim H. K., Kang J. S., Kim D. S., Chae H. J., You Y. O., Kwon K. B., Lee Y. R. (2013). Int. J. Mol. Med..

[cit11] Wang X., Zheng T., Kang J. H., Li H., Cho H., Jeon R., Ryu J. H., Yim M. (2016). Eur. J. Pharmacol..

[cit12] Kim M. H., Choi Y. Y., Lee H. J., Lee H., Park J. C., Yang W. M. (2015). J. Periodontal Implant Sci..

[cit13] Reynolds J. J., Meikle M. C. (1997). Periodontology 2000.

[cit14] Bartold P. M., Cantley M. D., Haynes D. R. (2010). Periodontology 2000.

[cit15] Fisher G. J., Datta S. C., Talwar H. S., Wang Z. Q., Varani J., Kang S., Voorhees J. J. (1996). Nature.

[cit16] Trindade F., Oppenheim F. G., Helmerhorst E. J., Amado F., Gomes P. S., Vitorino R. (2014). Proteomics: Clin. Appl..

[cit17] Hatipoglu H., Yaylak F., Gungor Y. (2015). Diabetes, Metab. Syndr. Obes.: Targets Ther..

[cit18] Cobb C. M. (2002). J. Clin. Periodontol..

[cit19] Smith P. C., Munoz V. C., Collados L., Oyarzun A. D. (2004). J. Periodontal Res..

[cit20] Santos J., La V. D., Bergeron C., Grenier D. (2011). J. Periodontal Res..

[cit21] Makela M., Salo T., Uitto V. J., Larjava H. (1994). J. Dent. Res..

