# Supplementary figures and images for: Angelica gigas ameliorates the destruction of gingival tissues via inhibition of MMP-9 activity
Source: RSC Adv. 2018 Apr 9;8(24):13089–93. doi: 10.1039/c7ra12531c (PMC9079679; doi:10.1039/c7ra12531c)

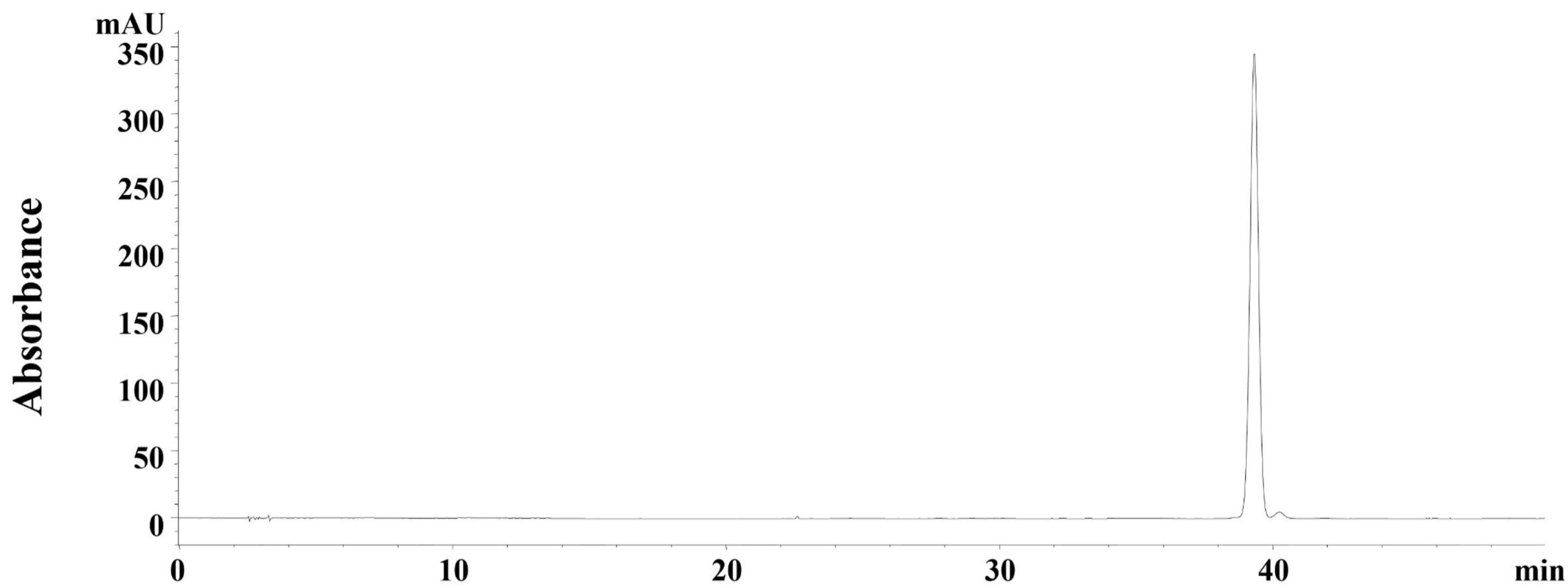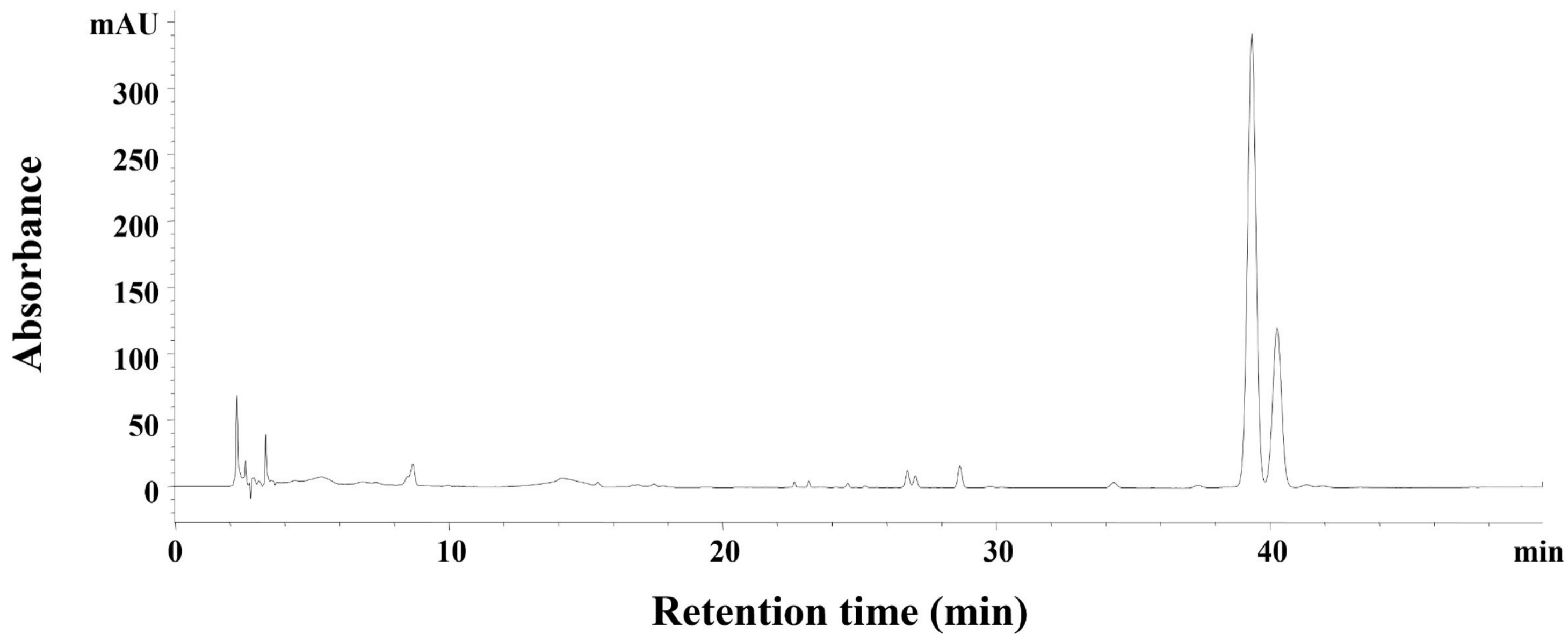

Supplement: RA-008-C7RA12531C-s001 [file RA-008-C7RA12531C-s001.pdf]
